# Lobomycosis in Offshore Bottlenose Dolphins (*Tursiops truncatus*), North Carolina

**DOI:** 10.3201/eid1504.081358

**Published:** 2009-04

**Authors:** David S. Rotstein, Leslie G. Burdett, William McLellan, Lori Schwacke, Teri Rowles, Karen A. Terio, Stacy Schultz, Ann Pabst

**Affiliations:** National Oceanic and Atmospheric Administration, Silver Springs, Maryland, USA (D.S. Rotstein, T. Rowles); University of Tennessee College of Veterinary Medicine, Knoxville, Tennessee, USA (D.S. Rotstein); Medical University of South Carolina, Charleston, South Carolina, USA (L.G. Burdett); University of North Carolina, Wilmington, North Carolina, USA (W. McLellan, A. Pabst); National Oceanic and Atmospheric Administration, Charleston (L. Schwacke); University of Illinois, Chicago, Illinois, USA (K.A. Terio, S. Schultz)

**Keywords:** Dolphin, Tursiops truncatus, Lacazia loboi, lobomycosis, granulomatous dermatitis, North America, North Carolina, dispatch

## Abstract

*Lacazia loboi*, a cutaneous fungus, is found in humans and dolphins from transitional tropical (Florida) and tropical (South America) regions. We report 2 cases of lobomycosis in stranded bottlenose dolphins (*Tursiops truncatus*) and 1 case of lobomycosis-like disease in 1 free-swimming, pelagic, offshore bottlenose dolphin from North Carolina, where no cases have previously been observed.

*Lacazia loboi* is a fungus (order Onygenales) that has not yet been cultured (*1*). Infection results in dermal and subcutaneous granulomas and 6–12-μm yeast-like bodies connected in chains by a small tubule (*2*); spread by the lymphatic system has been reported (*3*). Hematogenous spread and contiguous spread have not been excluded as means of propagation. Infections have been reported in humans (*4*) and dolphins, including Guiana dolphins (*Sotalia guianensis*) in tropical climates (Latin America) (*5*) and Atlantic bottlenose dolphins (*Tursiops truncatus*) in transitional tropical climates (Indian River Lagoon and Gulf of Mexico, Florida; Matagorda Bay, Texas; and Bay of Biscay, Europe) (*6*–*8*). We report 2 cases of lobomycosis in offshore (pelagic) bottlenose dolphins stranded off North Carolina in 2005 and 2008.

## The Cases

### KLC020

**Figure 1:**
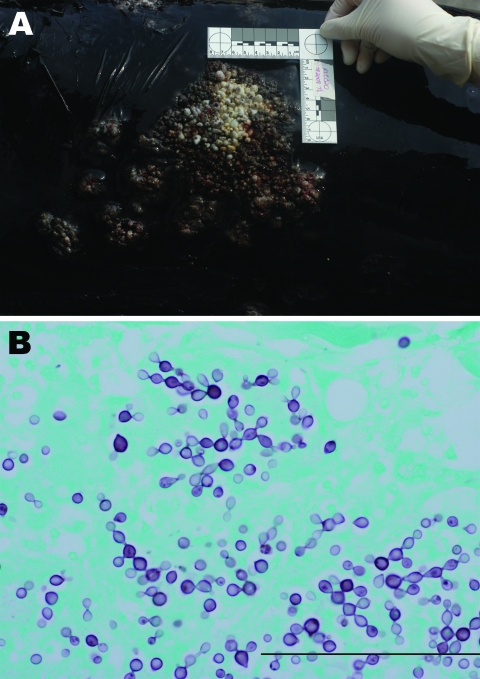
A) Serpiginous dermal nodules covering the dorsum of an offshore bottlenose dolphin (KLC020). B) Gomori methenamine silver–stained sections of dermis showing yeast-like structures connected by neck and arranged at various angles (magnification ×400). Scale bar = 100 μm.

On August 20, 2008, a dead male Atlantic bottlenose dolphin was found stranded on the North Carolina coast. On the basis of overall length, rostrum length, and flipper size, the dolphin was identified as belonging to the offshore ecotype (*9*). Gross findings included numerous serpiginous and coalescing, raised, ulcerated-to-papillary nodules on the dorsum anterior to the dorsal fin and extending to the mid-body (Figure 1, panel A). Other gross lesions included verminous pterygoid sinusitis and pneumonia and mild dermal and retroperitoneal cestodiasis.

Dermal and subcutaneous granulomas composed of multinucleated giant cells, epithelioid macrophages, lymphocytes, and plasma cells were present in all skin sections and surrounded yeast-like structures. Fungi (6–10 μm) were connected in chains to adjacent fungal bodies by a thin neck (Figure 1, panel B). Other findings included parasitic migratory tracts in the brain, parasitized lungs, and pterygoid sinuses.

DNA was isolated from fresh frozen skin samples (DNeasy Tissue Kit; QIAGEN, Valencia, CA, USA) and amplified by using 28S rRNA generic primers and MicroSeq D2 LSU rDNA primers (Applied Biosystems, Foster City, CA, USA). Amplicons were sequenced at the University of Chicago Cancer Sequencing Facility and were most closely (97%) related to *Paracoccidiodes brasiliensis*. *Paracoccidioides* spp., *Lacazia* spp., and *Emmonsia* spp. are related fungi; validated sequences are not available for amplified regions for *Lacazia* spp.

### AJW001

On March 5, 2005, a live male offshore Atlantic bottlenose dolphin was found stranded on Carolina Beach, North Carolina. The dolphin was in fair-to-good body condition. The most obvious gross finding was a few ulcerated dermal nodules scattered across the dorsum. Histologic findings from dermal nodules included granulomatous inflammation with numerous fungal yeast-like structures as in case KLC020. The dolphin also exhibited nonsuppurative meningoencephalitis, bronchointerstitial pneumonia, necrotizing hepatitis, and necrotizing adrenalitis associated with *Toxoplasma* spp.–like cysts and tachyzooites.

### Live Sighting

**Figure 2:**
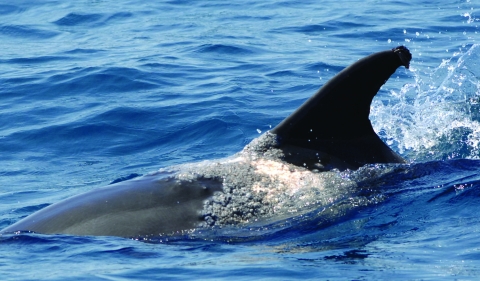
Free-swimming bottlenose dolphin (offshore ecotype) sighted off the Outer Banks of North Carolina with raised gray to white nodules over the dorsal surface, consistent with those of lobomycosis seen in other Atlantic bottlenose dolphins. *Xenobalanus* sp., a barnacle, is adhered to the tip of the dorsal fin. Image provided by Ari Friedlander, Duke University Marine Laboratory, Beaufort, NC, USA.

On May 26, 2008, a free-swimming offshore bottlenose dolphin was sighted by a vessel survey team from Duke University Marine Laboratory at 35.66584°N, 74.79782°W, ≈60 km off Oregon Inlet on the Outer Banks of North Carolina (Figure 2). The animal had a large region of raised epidermal gray to white nodules over the entire dorsal surface cranial to the dorsal fin. These lesions are consistent with those seen in the other bottlenose dolphins in this report and lesions seen on bottlenose dolphins from the Indian and Banana rivers in Florida (*10*). The location of this sighting suggests that this dolphin is of the offshore ecotype (*11**,**12*).

## Conclusions

Lobomycosis has been reported in the United States in coastal bottlenose dolphin populations in the Indian River Lagoon on the eastern coast of Florida, in the Gulf of Mexico off the western coast of Florida, and off the Texas Gulf coast. The Indian River Lagoon dolphin population has been assessed for temporal and spatial prevalence of lobomycosis (*10*). A prevalence of 6.8% was observed in that population from 1996 through 2006, and most cases were observed in the southern portion of this elongate body of water (*10*). The number of new cases per year, as determined from photograph identification studies, ranged from 1 to 9. Resolution of lesions has not been reported. In comparison, a prevalence of 3.9% was observed in a photograph identification study of *S*. *guianensis* from the Paranagua estuary in Brazil, a site of anthropogenic impact (*5*).

The cases in 2 stranded dolphins and in 1 photographed dolphin occurred in a subtropical climate of North America and involved an offshore rather than a coastal species. Information regarding unknown factors about these diseased animals, including host and pathogen range, pathogen molecular characterization, and environmental factors, could lead to a new range of organismal survival.

The offshore ecotype of bottlenose dolphins is generally found in waters >40 m deep but has been observed as close as 7.3 km from the coast at depths of 13 m (*12*). Bottlenose dolphins with offshore characteristics have been found as far south as the Florida Keys (*12*), but latitudinal movements of the offshore ecotype are not well understood. The 3 dolphins may have had a range that extended to the tropics where exposure could have occurred, rather than occurring in the region of stranding. A better understanding of movements of offshore ecotypes is needed so that potential exposure pathways can be inferred.

During preparation of this report, dorsal fin photographs from photograph identification projects along the mid-Atlantic coast identified 2 additional offshore *T*. *truncatus* that had skin lesions consistent with lobomycosis-like disease, a term used to describe gross observations that cannot be confirmed histologically (*5*). A more expansive study of photograph identification records could provide information on additional suspected cases. However, prevalence calculations from these studies will be negatively biased because the objective is generally to acquire photographs of the dorsal fin only. Dart biopsy of affected animals could confirm infection of dolphins with suspected cases but would require accuracy of sampling that may not be feasible in field conditions.

Because *L*. *loboi* has not been cultured, molecular techniques have been used to characterize the fungus. It is most related to the fungal order Onygenales, which includes *Emmonsia* spp. and *Paracoccioidies* spp. There are limited DNA sequences from *Lacazia* spp. for comparison, and PCR results for suspected *Lacazia* spp. are similar to those for *P*. *brasiliensis* (*13*), which are consistent with our findings for samples from case KLC020. The host range of *P*. *brasiliensis*, method of exposure (inhalation), and propensity for multisystemic invasion makes this fungi an unlikely causative agent of the disease in the 2 stranded *T*. *truncatus* dolphins. Histologic features of *P*. *brasiliensis* were also consistent with lobomycosis.

Decreased lymphocyte populations, which indicates decreased immune function, have been observed in animals with lobomycosis from the Indian River Lagoon (*14*) compared with noninfected cohorts in capture-and-release studies. Systemic disease has been reported in dolphins with lobomycosis from this lagoon (*15*). Of the 2 dolphins from North Carolina with lobomycosis, 1 had disseminated *Toxoplasmosis* spp.–like protozoal infection, and the other had suspected parasitic migration to the brain. Despite no histologic evidence of lymphoid depletion, lymphocytic function could not be determined for either animal. In stranded cetaceans such as dolphins, concurrent systemic disease is not an unexpected finding. Whether the presence of the fungus predisposes the animal to infectious processes, lowers immunity, or is a sign of decreased immunity may be best addressed in long-term capture-and-release studies in areas where the fungus is endemic and where data may be available from animals before and after infection.

Confirmation of lobomycosis in 2 stranded cetaceans off the coast of North Carolina represents a change in the northern distribution of this organism. Additional information on distribution and movements of offshore population(s) is needed to understand the prevalence and potential sources of infection.
